# Transcriptome Sequencing and Comparative Analysis of *Saccharina japonica* (Laminariales, Phaeophyceae) under Blue Light Induction

**DOI:** 10.1371/journal.pone.0039704

**Published:** 2012-06-27

**Authors:** Yunyan Deng, Jianting Yao, Xiuliang Wang, Hui Guo, Delin Duan

**Affiliations:** 1 Institute of Oceanology, Chinese Academy of Sciences, Qingdao, China; 2 Graduate University of Chinese Academy of Sciences, Beijing, China; East Carolina University, United States of America

## Abstract

**Background:**

Light has significant effect on the growth and development of *Saccharina japonica*, but there are limited reports on blue light mediated physiological responses and molecular mechanism. In this study, high-throughput paired-end RNA-sequencing (RNA-Seq) technology was applied to transcriptomes of *S. japonica* exposed to blue light and darkness, respectively. Comparative analysis of gene expression was designed to correlate the effect of blue light and physiological mechanisms on the molecular level.

**Principal Findings:**

RNA-seq analysis yielded 70,497 non-redundant unigenes with an average length of 538 bp. 28,358 (40.2%) functional transcripts encoding regions were identified. Annotation through Swissprot, Nr, GO, KEGG, and COG databases showed 25,924 unigenes compared well (E-value <10^−5^) with known gene sequences, and 43 unigenes were putative BL photoreceptor. 10,440 unigenes were classified into Gene Ontology, and 8,476 unigenes were involved in 114 known pathways. Based on RPKM values, 11,660 (16.5%) differentially expressed unigenes were detected between blue light and dark exposed treatments, including 7,808 upregulated and 3,852 downregulated unigenes, suggesting *S. japonica* had undergone extensive transcriptome re-orchestration during BL exposure. The BL-specific responsive genes were indentified to function in processes of circadian rhythm, flavonoid biosynthesis, photoreactivation and photomorphogenesis.

**Significance:**

Transcriptome profiling of *S. japonica* provides clues to potential genes identification and future functional genomics study. The global survey of expression changes under blue light will enhance our understanding of molecular mechanisms underlying blue light induced responses in lower plants as well as facilitate future blue light photoreceptor identification and specific responsive pathways analysis.

## Introduction

Light is a crucial environmental factor for growth and development of photosynthetic eukaryotes. Plants deploy sensory photoreceptors that assess and adapt to the quality and quantity of light fluctuations [1], [2]. Until now, known photoreceptor classes are the UVB photoreceptors [3], [4]; the red/far-red reversible photoreceptors, phytochromes PhyA-PhyE [5]; three blue UVA photoreceptor classes: cryptochromes (CRY1, CRY2 and CRY3) [6], [7], phototropins (PHOT1 and PHOT2) [8], aureochromes (AUREO1 and AUREO2) [9], [10]. Blue light (BL) photoreceptors govern cellular responses such as photoreactivation, plant development and circadian photoentrainment in bacteria, plants and animals [11]. In marine environment, BL is predominant because shorter and longer light wavelengths could not penetrate sea water mass [12]. There are many reports on BL mediated physiological responses in land plants [13], [14], but records on BL regulation of morphogenesis and life history in algae are few and only limited to documents of *Vaucheria* [15], [16], [17], [18], [19] and some brown algae [20], [21], [22].

*Saccharina japonica* (Areschoug) Lane, Mayes, Druehl and Saunders, is one of important commercial seaweed that naturally inhabits sublittoral zones where BL is predominant [23], [24]. Previous reports show BL stimulates *Saccharina* gametophyte growth and sporophyte reproduction [25], [26], [27], [28], [29], [30]. It is implied that BL photoreceptor is involved in the kelp growth and development [27], and hints that prevalent existence of BL photoreceptor in the stramenopiles, which including the Phaeophyceae, Xanthophyceae, Bacillariophyceae, Chrysophyceae and Raphidophyceae. In terms of phylogenetics, stramenopiles differ from green plants and possess new type of BL receptor. Recently the new type of BL receptor, AUREOs, is discovered in photosynthetic stramenopile members *Vaucheria frigida* (Xanthophyceae) and *Fucus distichus* (Phaeophyceae) [9], [10], and the conserved motifs of AUREOs are regarded as common and specific function of BL receptor in all stramenopiles [10]. Although BL-mediated physiological responses and morphogenesis changes have been observed in *Saccharina* [31], [32], the behavior of the BL receptor gene and its transcription analysis are far from understanding, especially our knowledge to *Saccharina* genome is limited.

High-throughput RNA-sequencing (RNA-Seq) provides new strategies for analyzing functional complexity of transcriptomes [33], [34], [35]. So far, it has been used to interrogate eukaryotic transcriptomes of yeast [36], [37], [38], mice [34], [39], humans [40], [41], [42], *Arabidopsis* [43], *Caenorhabditis elegans* [44], rice [45], *Vitis vinifera* [46], cucumber [47], *Lateolabrax japonicus* [48], maize [49], *Aspergillus oryzae* [50], large yellow croaker [51] and whitefly [52]. Compared with conventional transcriptome analysis approaches, it can quantify absolute gene expressions and provide more insight and accuracy than microarrays analysis [34], [35]. Furthermore, unlike hybridization-based approaches, RNA-Seq is not limited to detect transcripts that correspond to existing genomic sequence [33], which enable us more feasible to analysis organisms without genomic information.

In higher plants, light signals perceived by photoreceptors trigger dramatic transcriptome shifts that regulate growth and development [53]. While to algal materials, fewer reports are addressed on the transcriptiome analysis under the light treatment [54]. Expression profiling researches indicate that light induces profound gene expression changes in *Arabidopsis* [55], [55], [56], [57], [58,60], rice [58], [61] *Lotus japonicas* [62], and and *Ostreococcus tauri* [54]. These light-responsive genes include many transcripton factors and fall into various functional categories mainly involved in photomorphogenesis processes, circadian clock function, DNA repair, photosynthetic light reactions, photorespiration, photosynthetic carbon, metabolism and biosynthesis [53], [54], [55], [56], [57], [58], [59], [60], [61], [62].

For a broad testing the effects of BL induced physiological responses in *S. japonica*, RNA-Seq technology was applied to analysis the kelp transcription profile exposed to BL and darkness respectively, and the dynamic variation of transcriptome was interrogated. Our aim was to decipher transcriptomic changes and related genes behaviors under BL induction as well as verify BL receptor genes and the involved transduction pathway to the lower plants on the transcriptomic level.

## Materials and Methods

### Plant Material

Fresh juvenile sporophytes of *S. japonica* were collected from cultivated rafts in Rongcheng, Shandong, China in March, 2011. Healthy individuals were selected, rinsed with sterilized seawater for several times to remove epiphytes and cultured in constant darkness for 4 h. Washed materials were immersed in sterilized seawater under darkness and blue light for 2 h, respectively. Blue light-emitting diodes (LEDs) of wavelength 460–475 nm (Ichia, Shanghai, Japan) were used as light sources. Detected irradiances of 300–390 µmol photons m^−2 ^s^−1^ were measured with a quantum photometer (LI-COR, LI-250, Nebraska, USA). Cultures were carried out at 8±1.0°C. After washing with deionized distilled water, samples were dried with hygroscopic filter paper and frozen immediately in liquid nitrogen.

### Preparation of cDNA Library for RNA-Seq

For each treatment (darkness and BL exposed, respectively), about 100 g fresh algae materials (10 individuals) were mixed for RNA preparation. Total RNA was extracted according to the Yao et al. [63], and was treated with RNase-free DNase I (TaKaRa, Dalian, China) to remove residual genomic DNA. RNA integrity was confirmed via an Agilent Technologies 2100 Bioanalyzer with a minimum RNA integrated numerical value of 7. For each treatment, mRNAs were purified from the 20 µg total RNA using oligo (dT) magnetic beads and fragmented using fragmentation buffer. Cleaved short RNA fragments were used for first-strand cDNA synthesis using reverse transcriptase and hexamer-primer. Followed by second strand cDNA synthesis using DNA polymerase I and RNase H, cDNA fragments were selected for PCR amplification and cDNA library products were used for sequenced analysis via the Illumina HiSeq™ 2000.

### Transcriptome Analysis

Raw sequencing data were deposited in the GEO database at NCBI (accession number GSE33853). Raw reads were cleaned by removing adaptor sequences, empty reads and filtering reads containing unknown nucleotides (Ns) >5, and remaining clean reads were assembled into unigenes using SOAPdenovo [64]. TGICL [65] was used to acquire a single set of non-redundant unigenes. ESTScan [66] was used to analyze the coding sequences (CDs) of unigenes. All the non-redundant unigenes were used for blast search and annotation against the NCBI nr database, SwissProt database, Kyoto Encyclopedia of Genes and Genomes (KEGG) database and Cluster of Orthologous Groups (COG) database with 10^−5^ E-value cutoff. Functional annotation by gene ontology (GO) terms was analyzed using Blast2go program [67]. WEGO [68] was used to classify GO function.

### Identification of Differentially Expressed Genes

RPKM (reads per kilobase per million reads) were used to evaluate expressed value and quantify transcript levels [35]. P value and FDR (false discovery rate) were manipulated to determine differentially expressed unigenes [69]. Assuming that R differentially expressed genes have been selected, S genes really show differential expression, whereas the other V genes are false positives. If error ratio Q = V/R <5%, FDR should be ≤0.05. In the present study, unigene, P≤0.05, FDR ≤0.001, absolute value of log_2_Ratio ≥1 and unigene length ≥500 bp were used as thresholds to assess the different significance of gene expression. For pathway enrichment analysis, all differentially expressed unigenes were mapped to terms in KEGG database and searched for significantly enriched KEGG terms compared to the whole transcriptome background.

### Quantitative Real-time PCR Validation

A total of 11 representative BL response-relevant unigenes (BL receptor, ZTL/FKF1/LKP2, CK2α, APR 5/APR 7/APR 9, polyketide synthase, COP 9 signalosome complex subunit, DET1 and photolyase homologues) generated by RNA-seq were selected for experimental validation. Real-time quantitative PCR was performed with the SYBR® *Premix Ex Taq*™ (TakaRa, Tokyo, Japan) on the Takara TP800 Thermal Cycler Dice™ (Takara). First-strand cDNA was synthesized from 2 µg of total RNA as described above and used as a template for real-time PCR with specific primers (File S1). β-actin fragment amplification of *S. japonica* was used as internal control tests. Real-time PCR was performed in volume of 25 µl, and cycling conditions were 95°C for 30 s, followed by 40 cycles of 95°C for 5 s, 50°C for 30 s and 72°C for 30 s. All reactions were performed in biological triplicates, and the results were expressed relative to the expression levels of β-actin in each sample by using the 2ΔΔCT method.

## Results and Discussion

### Raw Reads Processing and Assembly

For the comparisons, two cDNA treatments prepared from dark and BL exposure respectively was sequenced with the Illumina sequencing platform. Raw reads was transformed by base calling from image data output from sequencing machine. After removing adapters and unknown or low quality bases, approximately 25.32 and 23.96 million clean reads were obtained (File S2). SOAPdenovo [64] was used to assemble clean reads into contigs in which the longest assembled sequences without N. Mapping reads to contigs and combining paired-end information created scaffolds and unknown bases were filled with Ns. After filling gaps in the scaffolds, 83,194 and 56,934 unigenes were generated from darkness and BL exposed treatments. Then removing partial overlapping sequences using CAP3 [70] yielded 70,497 non-redundant unigenes (Table 1). These sequences provided abundant information to further analyze the BL-related genes in *S. japonica.*

**Table 1:** Distribution of assembled unigenes.

Length (bp)		Total Number		Percentage
100–500		47,293		67.09%
500–1000		15,040		21.33%
1000–1500		4,638		6.58%
1500–2000		1,911		2.71%
≥2000		1,615		2.29%
Total		70,497		
				
	Total Length (bp)		37,895,389	
	N50		679	
	Mean		538	

N50  =  median length of all unigenes.

Mean  =  average length of all unigenes.

### Annotation of Non-redundant Unigenes

To understand the transcriptome of *S. japonica*, we annotated the unigene sets based on sequence homologies to annotated sequences and identified conserved protein domains in other species. ESTscan software analysis showed about 28,358 (40.2% of all distinct unigenes) have reliable coding sequences (CDs) [66]. CD-containing unigenes have high potential for translation into functional proteins and most translated to proteins with >100aa. Comparison with the Nr, Swissprot, KEGG, GO databases established 25,924 unigenes that compared well with known gene sequences (Table 2 and File S3).

**Table 2:** Annotation of non-redundant unigenes.

Database	Number of annotated unigenes	Percentage of annotated unigenes
Swissprot	15,281	53.89%
Nr	24,514	86.44%
GO	10,440	36.82%
KEGG	8,476	29.89%
COG	9,650	34.03%

All 28,358 CD-containing unigenes revealed by ESTscan were annotated though Swissprot, Nr, GO, KEGG, and COG databases. 25,924 unigenes amongt them compared well (E-value <10^−5^) with known gene sequences in existing species.

**Figure 1:**
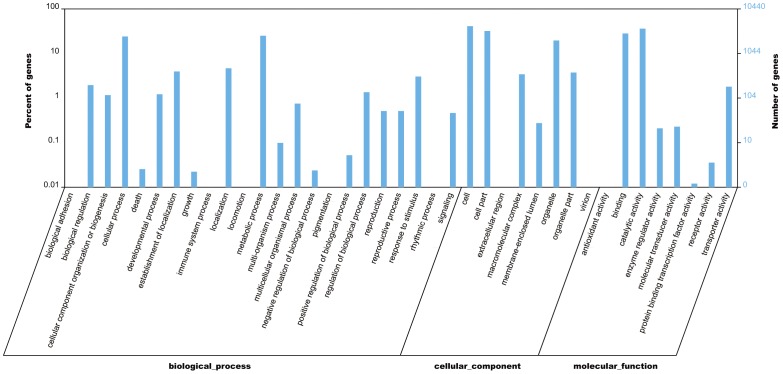
GO annotation of non-redundant unigenes. Good hits were aligned to the GO database, and 10,440 transcripts were assigned to at least one GO term. All the unigenes were grouped into three major functional categories, namely biological process, cellular component, and molecular function. The right y-axis indicates the number of unigenes in a category. The left y-axis indicates the percentage of a specific category of unigenes in that main category.

GO (Gene Ontology) assignments [71] were applied to classify functions of predicted *S. japonica* unigenes. A total of 10,440 sequences were assigned at least one GO term (Figure 1), among which 6,051 were assigned at least one GO term in the biological process category, 5,460 in the cellular component category and 8,906 in the molecular function category. These unigenes were further classified into functional subcategories. Sequences with GO terms corresponding to the “biological process” group were divided into 24 subcategories, “cellular component” into 8 subcategories, and “molecular function” into 8 subcategories. The largest subcategory found in the “biological process” group was “metabolic process” which comprised 29.1% of the unigenes in the subcategory. In the “cellular component” and “molecular function” categories, “cell” and “catalytic activity” were the most abundant GO terms, making up 36.1% and 50.4% of each subcategory, respectively. In addition, there were high percentages of unigenes in the categories “cell part,” “binding,” “cellular process” and only a few unigenes in “biological adhesion”, “locomotion”, “rhythmic process” and “extracellular region.”

**Figure 2:**
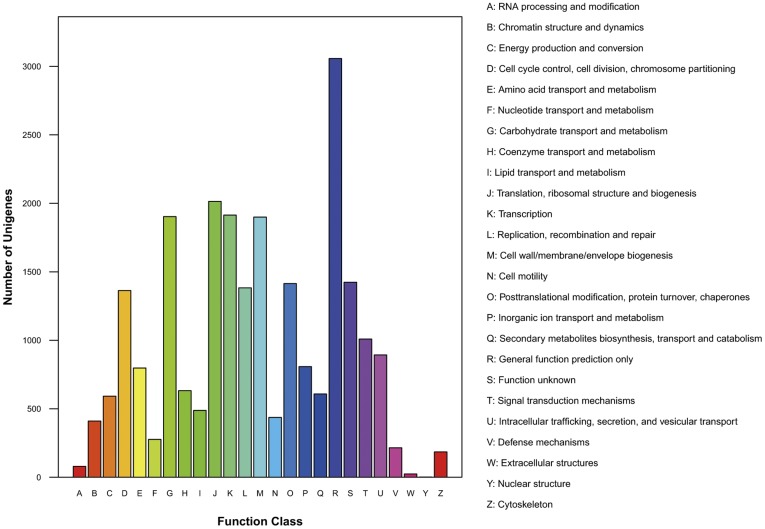
Histogram presentation of clusters of orthologous groups (COG) classification. A total of 9,650 sequences have a COG classification among the 25 categories.

To further evaluate the completeness of the transcriptome library and the effectiveness of annotations, we searched annotated sequences for genes involved in COG classifications [72]. COG annotation yielded approximately 9,650 putative proteins in 25 categories (Figure 2). Among those categories, the cluster for “General function prediction” was the largest group (3057, 12.8%), followed by “Translation, ribosomal structure and biogenesis” (2014, 8.5%) and “Transcription” (1914, 8.0%). Clusters for “Nuclear structure” (2, 0.008%), “Extracellular structures” (24, 0.101%) and “RNA processing and modification” (79, 0.332%) were the smallest groups.

Kyoto Encyclopedia of Genes and Genomes (KEGG) database [73] was used to identify the biological pathways in *S. japonica.* A total of 8,476 unigenes were mapped to 114 KEGG pathways. The pathways with most representation by the unique sequences were metabolic pathways (1903 members); spliceosome (901 members) and biosynthesis of secondary metabolites (771 members). These KEGG annotations provided a valuable resource for investigating specific gene functions and pathways in *Saccharina* and strongly supported future kelp genome annotation.

### Detection of BL Response-relevant Gene Sequences

For further insight into BL response in *S. japonica*, response-relevant gene sequences were analyzed. A total of 130 responsive unigenes sequences were obtained, among them 43 were putative BL photoreceptor candidates (Table 3 and File S4), of which 24 unigenes were homologous to known BL photoreceptor genes in higher plant or other algae, including cryptochrome, phototropin and aureochrome. One unigene was homologous to BL photoreceptor gene of bacteria (*Listeria innocua*). These sequences will certainly facilitate further BL photoreceptor genes identification in *Saccharina.*

**Table 3:** Blue light receptor genes/homologues in *S. japonica.*

Blue light receptorgene catalog	The number of unigenes	Homologs number in other species
Cryptochrome	7	7
Phototropin	26	7
Aureochrome	9	9
BL receptor in bacteria	1	1

In addition, 87 other unigenes were found to be homologous to the known BL response-relevant genes (File S5), which are essential components in physiological processes of circadian rhythm (clock-associated PAS protein ZTL, flavin-binding kelch repeat F-box protein 1 (FKF1), LOV kelch protein 2 (LKP2), circadian clock associated protein 1 (CCA1), LHY (LATE ELONGATED HYPOCOTYL), *Arabidopsis* pseudo-response regulator (APR 3, APR 5, APR 7 and APR 9), CK2α (casein kinase 2, alpha polypeptide), CK2β (casein kinase 2, beta polypeptide), serine/threonine-protein kinase WNK1, zinc finger protein CONSTANS (CO)), flavonoid biosynthesis (polyketide synthase, dihydroflavonol reductase, flavonoid hydroxylase, chalcone isomerase), photoreactivation (photolyase, DNA damage-binding protein, DDB1- and CUL4-associated factor, DET1 (de-etiolated 1)), and photomorphogenesis (COP 9 signalosome complex subunit, DET1). Future molecular and functional characterizations of these candidate genes could help to global identification of BL responsive genes and markers in algae.

### Global Changes in Gene Expression under BL

To characterize the differences of molecular response between the dark and BL treatments, unigene expression levels were calculated by RPKM using the formula [35]: RPKM = (10^9^ ×C)/(N × L), where C is the number of reads that uniquely aligned to the gene, N is the total number of reads that uniquely aligned to all genes, L is the sum of the gene in base pairs. The RPKM method eliminates the influence of gene length and sequencing discrepancy in calculating gene expression, allowing direct comparison of gene expression between treatments. Based on RPKM values, 11,660 differentially expressed unigenes (with P value <0.05, FDR ≤0.001, fold change value >2 and unigene length ≥500 bp) were indentified (File S6), including 7,808 upregulated and 3,852 downregulated unigenes. The large amount of regulated genes (17%) encountered here was in contrast to what has been recorded in *Arabidopsis*, where the proportion of significantly light or BL modified genes generally ranges from 1% to 5% [55], [56]. The dramatic expression profile suggested significant transcriptional complexities in *S. japonica* and its extensive transcriptome re-orchestrated during BL induction.

### Functional Annotation of Differentially Expressed Unigenes

**Figure 3:**
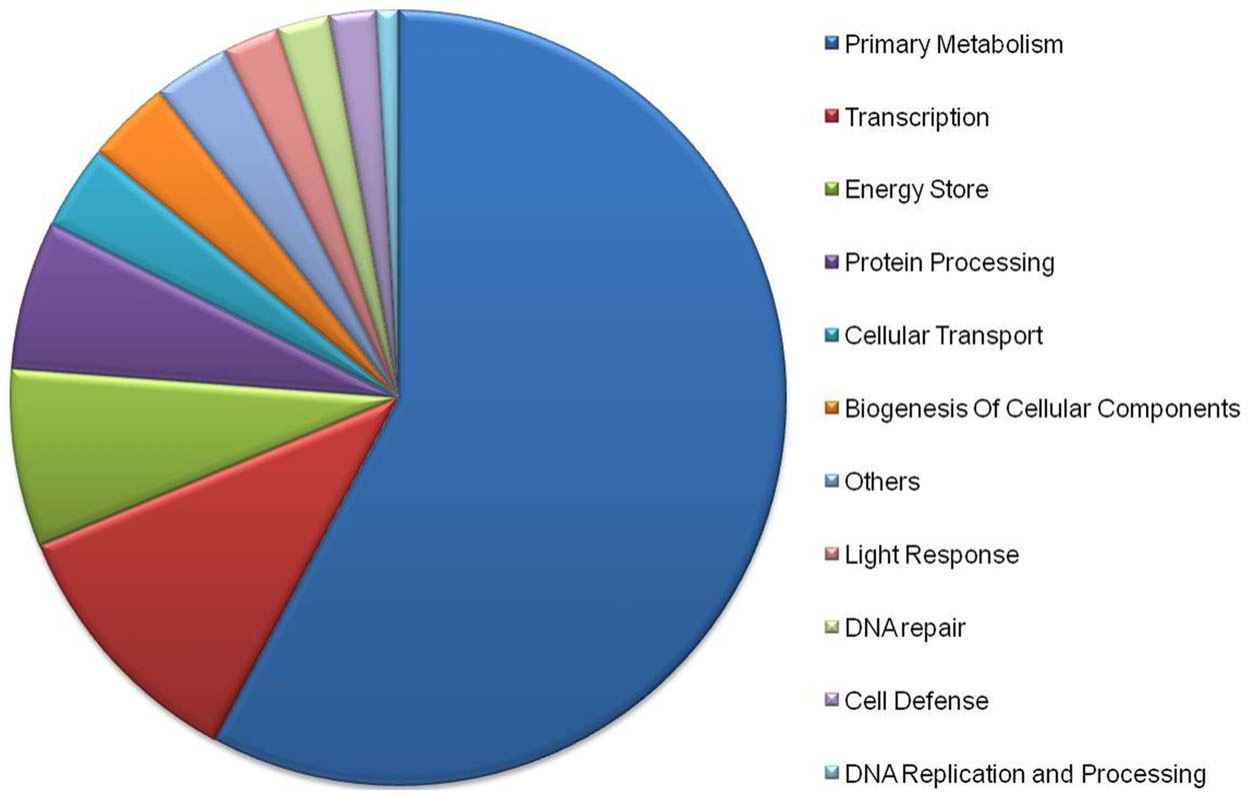
KEGG functional analyses of the differentially expressed unigenes (data from File S7).

All the differentially expressed sequences were mapped to KEGG database terms and compared with the whole transcriptome data, with a view to finding unigenes concerning metabolic or signal transduction pathways that were significantly enriched. Of 8476 unigenes with KEGG annotation, 4671 differentially regulated unigenes were identified between the two treatments. The other 6989 changed unigenes failed to match sequences in the current database and therefore represented potentially novel BL responsive genes. The three-fifths regulated genes functions were unknown, underlined molecular mechanisms underlying BL responses in lower plants were far from thoroughly understanding. Pathway enrichment analysis revealed that the annotated changes were mainly involved in primary metabolism, transcription, protein processing, cellular transport, biogenesis of cellular components, energy storage, light response and DNA repair (Figure 3 and File S7). These processes included biological pathways that directly or indirectly participated in response, and again reflected the large scale re-orchestrated during short-term acclimation to BL exposure. Some significantly prominent pathways were shown in Table 4.

**Table 4:** Significantly enriched pathways of differentially expressed unigenes.

Pathway category	Unigenes No.	%	Q-value
Plant-pathogen interaction	174	3.73	0.00000019
ABC transporters	77	1.65	0.00148163
Ribosome	129	2.76	0.01365288
Alanine, aspartate and glutamate metabolism	61	1.31	0.01896333
alpha-Linolenic acid metabolism	29	0.62	0.06159647

Unigenes No. and % indicate the number and the percentage of unigenes in each pathway from 4671 differentially expressed unigenes mapped to KEGG respectively.

**Figure 4:**
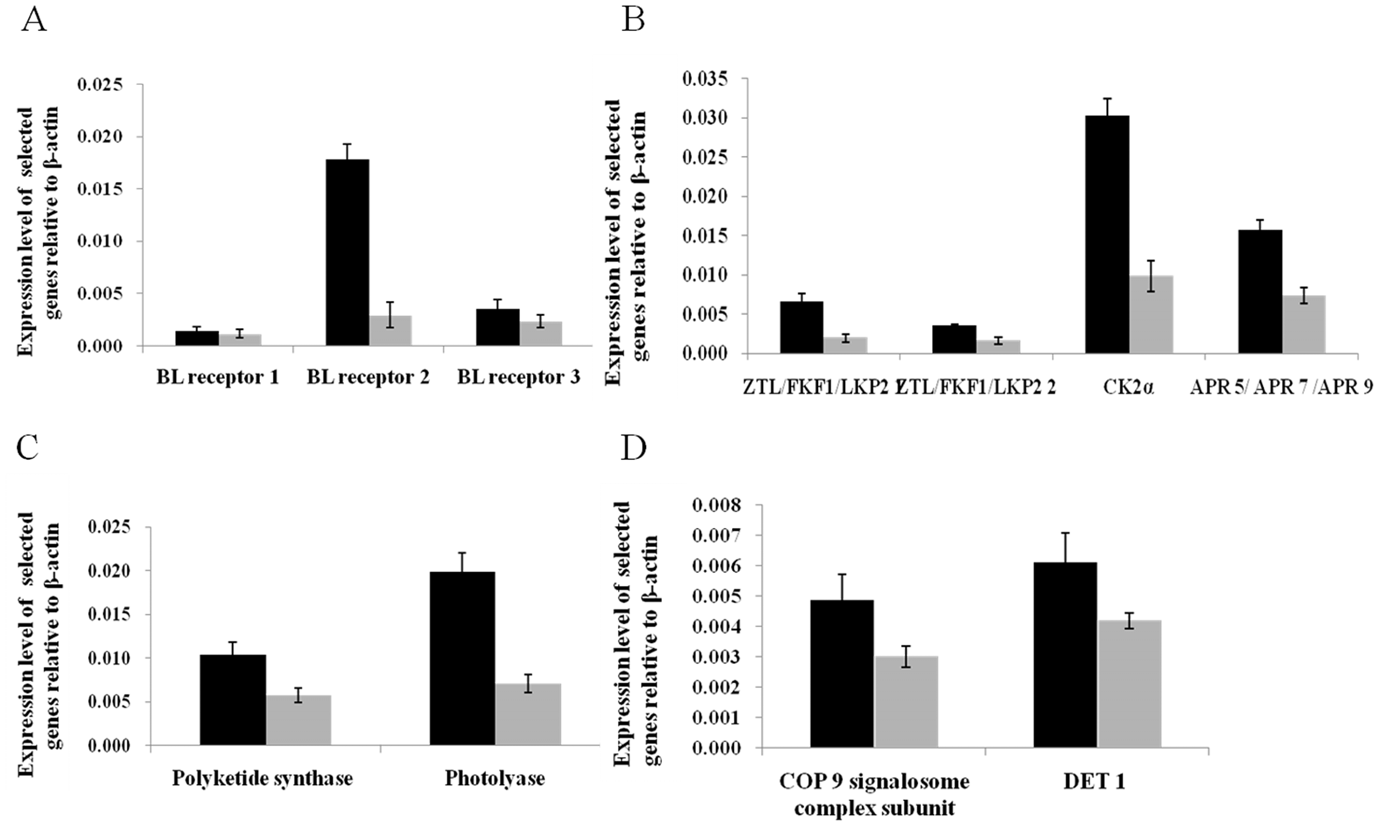
The expression analysis of selected genes from the RNA-seq by relative quantitative real-time PCR. Total RNA was extracted from *S. japonica* exposed to BL and darkness, respectively. Real-time PCR was used to validate gene expression changes of putative BL photoreceptors (A) and in pathways of circadian rhythm (B), flavonoid biosynthesis and photoreactivation pathways (C), and photomorphogenesis (D). Increases and decreases in relative levels of transcripts with respect to the control 18 S gene are shown. For each gene, the black bar indicates the gene expression ratio of kelp exposed to BL; the grey bar indicates the expression ratio of kelp exposed to darkness. Values are mean ± standard deviation.

The extensive transcriptome changes as observed inevitably demanded a multitude of signals for coordination. BL photoreceptor was one of the most possible triggers in the network. As BL activated receptor, it initiated BL signal transduction through the coordinated activation and repression of specific genes and regulated several downstream signaling pathways [6], [7], [8], [9], [10], [11]. In our study, among the 43 hypothetic BL photoreceptor unigenes (File S4), 28 sequences was highly elevated regulated after BL exposure (File S8), of which 6 genes was upregulated more than 10 folds. Increased expression of BL receptor genes was also observed by the real-time PCR (Figure 4A). Since very little information were known on the signal cascades and the relative pathway of BL sensing in algae, these sequences provided important clues for screening putative BL receptors genes and relative transcriptional factors. Besides, 34 BL-specific responsive genes (except the putative BL photoreceptor unigenes) were either found or recognized as important role during the algal circadian rhythm, flavonoid biosynthesis, photoreactivation and photomorphogenesis. We then focused our discussion on these physiological processes and highlighted emerging insights regarding information provided by the regulated sequences.

**Figure 5:**
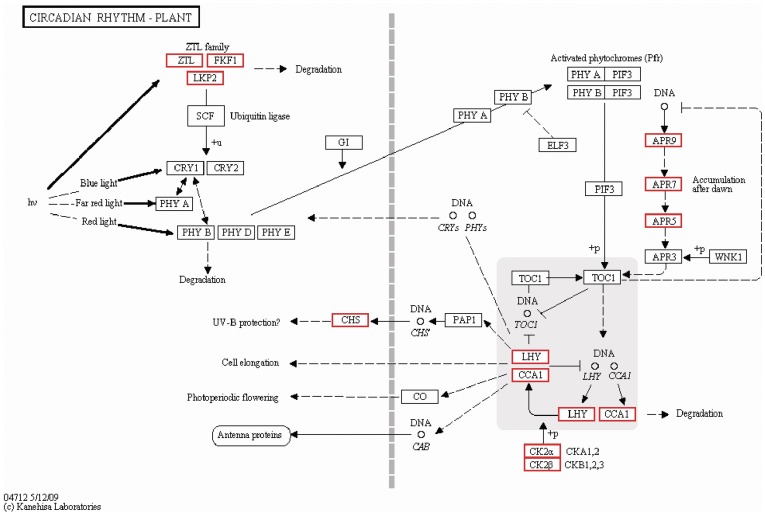
The circadian rhythm signal network in plant. Red color represents up-regulated unigenes after blue light exposure in our study.

The circadian rhythm is the temporal oscillation of genetic, metabolic and physiological processes based on the 24 h cycle. It is shaped by alternating day and night cycles and driven through an endogenous timekeeping mechanism [74], [75]. Previously, tremendous progress has been made in the molecular mechanisms in *Arabidopsis thaliana* [61], and some deduced proteins related to control TOC1 (TIMING OF CAB EXPRESSION 1) that functioned in constant darkness were considered as key determinants in circadian period of higher plants (Figure 5). To our yielded data, the increased abundances of transcripts of these proteins, including ZTL/FKF1/LKP2, CCA1/LHY, APR 5/APR 7/APR 9, CK2α and CK2β were detected (Figure 5 and File S9). Quantitative PCR results also demonstrated the upregulation of genes of ZTL/FKF1/LKP2, APR 5/APR 7/APR 9 and CK2α (Figure 4B). Among the total 20 highly expressed transcripts, 13 of them were homologue to ZEITLUPE family of putative BL photoreceptor, which consists of PAS-like LOV domain, F box domain and kelch repeats. LOV domain is the molecule responsible for flavin binding in known BL photoreceptors, while the F box motif is found in specific target substrates for proteolytic degradation [76]. In this case, upon BL activation, ZTL as component of SCF (Skp/Cullin/F-box) E3 ubiquitin complex recruits TOC1 for post-translational proteasomal degradation [76], [77], [78]. Other 5 unigenes, putative CCA1/LHY or CK2α/CK2β orthologue, were involved in CCA1/LHY mediated transcriptional repression of TOC1. CCA1/LHY as negative regulator activated by CK2 (Casein Kinase II) could bind with the TOC1 promoter to repress TOC1 expression [79], [80], [81]. The other 2 unigenes were paralogues to TOC1 relatives, APR5/APR7/APR9, which are important components for photoperiodic timekeeping and positively regulated by CCA1/LHY proteins [82], [83]. To our data obtained, 18 of the 20 regulated clock unigenes belonged to the core feedback loop, suggested the BL effects on the kelp clock were predominantly mediated via this part of the oscillator mechanism. In addition, we compiled 48 clock-associated coding sequences in *Saccharina*, which appeared to be orthologous or homologous to the *Arabidopsis* counterparts. A considerable conservation of some elements in circadian rhythm seems exist between *Saccharina* and *Arabidopsis*. Further verification of those homologous sequences is expected to not only deeper understand the kelp photoperiodism properties but also enrich knowledge on molecular mechanism of circadian rhythm in lower plants.

We also noticed elevated transcript abundance of polyketide synthase (CHS) (Figure 4C) and dihydroflavonol reductase (DFR) (File S10), which are two key enzymes in flavonoid biosynthesis pathway. In higher plants, CHS catalyzes the first committed step in the flavonoid biosynthesis and DFR is the first enzyme leading to anthocyanidins production. Their expression stimulated by UV/BL were considered to be protective mechanism as which promoted the accumulation of UV-absorbing flavonoids [84]. Here BL stimulated transcription of key enzymes in flavonoid biosynthesis corresponded to that the precious reports on UV/BL induction of flavonoid synthesis in higher plants [84], [85]. *Saccharina* naturally inhabit the sublittoral zone, which of rapid changing physical conditions when tides in and off. It is required to exhibit tolerance to various abiotic stressors such as osmotic pressure, temperature and water currents, and the flavonoids might play roles in stresses or UV protection in kelp. It is suspected that UV/BL increases the biosynthesis of flavonoids, which in turn, function in stresses or UV protection in *S. japonica*, future experiments are required to test this hypothesis.

Photoreactivation is a repair process of DNA pyrimidine dimers that results from UV-B light exposure [86]. It is catalyzed by BL dependent enzymes photolyases [87]. In our study, 16 transcripts of genes implicated in photoreactivation were detected (File S5), and 9 sequences of them exhibited different expression under BL exposure (File S11). One sequence (Unigene7340) homologous to photolyase was upregulated 2.8 fold after BL induction in our quantitative PCR, which was consistent with its nearly 2 folds elevated expression in RNA-seq (Figure 4C). The other 8 sequences including genes encoding DDB1- and CUL4-associated factor, DET 1 (de-etiolated 1) and DNA damage-binding protein. DET1 is a nuclear protein conserved to higher plants. In *Arabidopsis*, DET1 associated with factors of the poly-ubiquitination pathway (such as CUL4) and with the DNA repair pathway via DDB1 [88], [89]. Previously, photoreactivation have mainly been studied in virus, bacteria, fungi and higher plant, and very few works focused on algae. Whitaker first reported *Fucus furcatus* Gardner on the reactivation of UV inhibited rhizoid formation [90]. Followed damaging UV effects on *Acetabularia*, *Alaria* and *Saccharina* (*Laminaria*) photoreactivated were recorded, and BL was higher effective than white, green or red light in these processes [91], [92], [93]. Han and Kain inferred a BL absorbing photolyase was involve in the BL induced reactivation of UV-irradiated damages in brown algae [92], [93]. Our results confirmed their deduction, and suggested that the DNA damage genes were not only triggered by UV exposure, but also responded to BL. The 9 upregulated unigenes were extremely related to the early stage of the BL mediated DNA repair in juvenile sporophytes of *S. japonica*.

Photomorphogenesis is a serial of developmental changes in growth and differentiation upon the exposure to light [1]. In higher plants, CRYs are the mainly BL photoreceptors involved in the process [86], [87]. Here we encountered 5 unigenes associated with kelp photomorphogenesis (File S5). One of them (Unigene 48767) was found to be homolog of DET1, a photomorphogenesis repressor, controls several genes in darkness in higher plant [94]. The other 4 sequences were homologous to subunit of COP9 signalosome complex, which is another repressor of photomorphogenesis in *Arabidopsis* [95]. As a component of ubiquitin-proteasome pathway, COP9 signalosome complex participates in targeted degradation of key transcription factors that regulates the photoresponsive genes expression [1]. HY5, a constitutive nuclear bZIP transcription factor, function positively in photomorphogenic development by binding to the promoters of light-inducible genes, is a primary target of this pathway. Previous studies showed that COP9 complex was highly conserved in mammals and higher plants [95], [96]. Our results indicated that it was also conserved in algal phylum. However, no other homolog of signaling components in higher plants photomorphogenesis was identified in our data set, suggesting poor conservation of photomorphogenic basic elements existed between the lower plant-kelp and higher plant. Besides, the DET1 sequence and one COP9 subunit homology were prominent expressed in the BL relative to dark treatment (File S12). Our quantitative PCR analysis also confirmed differential expression of the two unigenes (Figure 4D), suggested that the two repressors might play important role in the photomorphogenesis of juvenile sporophyte.

### Conclusion

This study investigated the transcriptome profile of BL-exposed *S. japonica* using Illumina RNA-seq technology to identify responsive genes and specific pathways involved in BL response of kelp. Although current knowledge was limited by the poorly annotated kelp genes and scanty reports of BL-mediated physiological responses in algae, we identified 43 putative BL photoreceptor unigenes and simply elucidated 4 BL specific responsive functions in the BL induced gene set. The present assessment of transcriptome and gene expression in *S. japonica* included the most comprehensive sequence resource yet available for the species lack of genome information. Our results provided important clues for further BL photoreceptor and other functional genes identification in kelp as well as paved the way for more details investigations of mechanisms underlying the 4 BL specific responsive pathways in the lower plants.

## Supporting Information

File S1 Primers for relative quantitative realtime PCR. Primers were designed from the sequences of the *S. japonica* transcriptome library by using Primer Premier 5.0.
(XLS)

File S2 Overview of output statistics on *S. japonica* transcriptome sequencing.
(DOC)

File S3 Details on 25,924 unigenes annotated in the transcriptome of *S. japonica*.
(XLS)

File S4 Details on 43 blue light-receptor genes/homologues in *S. japonica*.
(XLS)

File S5 Summary of 87 blue light response-relevant genes/homologues in *S. japonica*.
(XLS)

File S6 11,660 differentially expressed unigenes between blue light and dark exposed samples.
(XLS)

File S7 KEGG functional analysis of the differentially expressed unigenes.
(XLS)

File S8 28 significant differentially expressed BL-receptors unigenes in *S. japonica*.
(XLS)

File S9 20 significant differentially expressed unigenes in circadian rhythm pathway in *S. japonica*.
(DOC)

File S10 3 significant differentially expressed unigenes in flavonoid biosynthesis pathway in *S. japonica*.
(DOC)

File S11 9 significant differentially expressed unigenes related to blue light induced photoreactivation in *S. japonica*.
(DOC)

File S12 2 significant differentially expressed unigenes related to blue light induced photomorphogenesis in *S. japonica*.
(DOC)
