# Supplementary material for: Transcriptome Sequencing and Comparative Analysis of Saccharina japonica (Laminariales, Phaeophyceae) under Blue Light Induction
Source: PLoS One. 2012 Jun 27;7(6):e39704. doi: 10.1371/journal.pone.0039704 (PMC3384632; doi:10.1371/journal.pone.0039704)
Supplement: File S2 — Overview of output statistics on S. japonica transcriptome sequencing. (DOC) [file pone.0039704.s002.doc]

**Table S2 Overview of output statistics on *S. japonica* transcriptome sequencing**

| **Sample** | **Darkness exposed** | **Blue light exposed** |
| --- | --- | --- |
| The number of total clean reads | 25,315,304 | 23,963,660 |
| Total base pairs (bp) of clean reads | 2,278,377,360 | 2,156,729,400 |
| Q20 percentage | 89.80% | 88.75% |
| N percentage | 0% | 0% |
| GC percentage | 52.13% | 54.72% |

Q20 percentage = proportion of nucleotides with quality value larger than 20

N percentage = proportion of unknown nucleotides in clean reads
