# Supplementary material for: Transcriptome Sequencing and Comparative Analysis of Saccharina japonica (Laminariales, Phaeophyceae) under Blue Light Induction
Source: PLoS One. 2012 Jun 27;7(6):e39704. doi: 10.1371/journal.pone.0039704 (PMC3384632; doi:10.1371/journal.pone.0039704)
Supplement: File S9 — 20 significant differentially expressed unigenes in circadian rhythm pathway in S. japonica. (DOC) [file pone.0039704.s009.doc]

***File S9 20 significant differentially expressed unigenes in circadian*** ***rhythm (plant) pathway in S. japonica***

| **Gene ID** | **Description** | **Fold** | **P value** |
| --- | --- | --- | --- |
| Unigene63416 | ZTL/FKF1/LKP2 | 11.7948 | 3.27E-04 |
| Unigene46429 | ZTL/FKF1/LKP2 | 3.2076 | 2.57E-06 |
| Unigene47495 | ZTL/FKF1/LKP2 | 13.6307 | 4.50E-49 |
| Unigene10687 | ZTL/FKF1/LKP2 | 3.3452 | 1.38E-59 |
| Unigene11320 | ZTL/FKF1/LKP2 | 2.5772 | 0 |
| Unigene14139 | ZTL/FKF1/LKP2 | 1.7123 | 1.87E-11 |
| Unigene19749 | ZTL/FKF1/LKP2 | 1.9765 | 1.74E-08 |
| Unigene70482 | ZTL/FKF1/LKP2 | 4.4674 | 3.41E-180 |
| Unigene21263 | ZTL/FKF1/LKP2 | 1.6089 | 6.51E-13 |
| Unigene49100 | ZTL/FKF1 | 4.2015 | 3.99E-18 |
| Unigene35321 | ZTL/FKF1 | 4.1443 | 3.35E-09 |
| Unigene47823 | ZTL/FKF1 | 6.5859 | 1.43E-55 |
| Unigene69770 | ZTL/FKF1 | 13.6025 | 4.63E-26 |
| Unigene11759 | CCA1/LHY | 2.2728 | 7.48E-34 |
| Unigene10312 | CCA1/LHY | 2.3132 | 4.83E-11 |
| Unigene9399 | CK2α | 1.8105 | 1.92E-187 |
| Unigene61208 | CK2α | 0.9346 | 2.00E-01 |
| Unigene48109 | CK2β | 2.3753 | 4.30E-11 |
| Unigene44659 | APR 5/ APR 7 /APR 9 | 3.0722 | 1.03E-08 |
| Unigene46535 | APR 7 | 4.0221 | 1.12E-04 |

Limitations of all differentially expressed unigenes are based on P value < 0.05 and FDR ≤ 0.001which indicated the unigene was significantly altered after BL exposure. The absolute value of “Fold” means the magnitude of up- or downregulation for each unigene after BL exposure; “+” indicates upregulation and “-” indicates downregulation.
