# Supplementary material for: Transcriptome Sequencing and Comparative Analysis of Saccharina japonica (Laminariales, Phaeophyceae) under Blue Light Induction
Source: PLoS One. 2012 Jun 27;7(6):e39704. doi: 10.1371/journal.pone.0039704 (PMC3384632; doi:10.1371/journal.pone.0039704)
Supplement: File S10 — 3 significant differentially expressed unigenes in flavonoid biosynthesis pathway in S. japonica. (DOC) [file pone.0039704.s010.doc]

**File S10 3 significant differentially expressed unigenes in flavonoid biosynthesis pathway in *S. japonica***

| **Gene ID** | **Description** | **Fold** | **P value** |
| --- | --- | --- | --- |
| Unigene26912 | Polyketide Synthase III | 2.1345 | 4.80E-229 |
| Unigene42303 | Polyketide Synthase III | 6.6873 | 4.99E-177 |
| Unigene25545 | dihydroflavonol-4-reductase | 1.2646 | 4.27E-11 |

Limitations of all differentially expressed genes are based on P value < 0.05 and FDR ≤ 0.001which indicated the unigene was significantly altered after BL exposure. The absolute value of “Fold” means the magnitude of up- or downregulation for each gene/homolog after BL exposure; “+” indicates upregulation and “-” indicates downregulation.
