# Supplementary material for: Transcriptome Sequencing and Comparative Analysis of Saccharina japonica (Laminariales, Phaeophyceae) under Blue Light Induction
Source: PLoS One. 2012 Jun 27;7(6):e39704. doi: 10.1371/journal.pone.0039704 (PMC3384632; doi:10.1371/journal.pone.0039704)
Supplement: File S11 — 9 significant differentially expressed unigenes related to blue light induced photoreactivation in S. japonica. (DOC) [file pone.0039704.s011.doc]

**File S11 9 significant differentially expressed unigenes related to blue light induced photoreactivation in *S. japonica***

| **Gene ID** | **Description** | **Fold** | **P value** |
| --- | --- | --- | --- |
| Unigene7340 | deoxyribodipyrimidine photolyase | 1.8222 | 1.17E-04 |
| Unigene16340 | DNA damage-binding protein | 2.727 | 2.28E-143 |
| Unigene68694 | DNA damage-binding protein | 3.635 | 6.93E-09 |
| Unigene13913 | DNA damage-binding protein | 1.0618 | 1.08E-04 |
| Unigene17807 | DDB1- and CUL4-associated factor | 2.4669 | 3.12E-62 |
| Unigene34020 | DDB1- and CUL4-associated factor | -1.2804 | 4.46E-05 |
| Unigene53446 | DDB1- and CUL4-associated factor | 12.4453 | 8.54E-05 |
| Unigene239 | DDB1- and CUL4-associated factor | 1.5772 | 7.31E-62 |
| Unigene48767 | DET 1 (de-etiolated 1) | 2.8415 | 9.07E-18 |

Limitations of all differentially expressed unigenes are based on P value < 0.05 and FDR ≤ 0.001which indicated the unigene was significantly altered after BL exposure. The absolute value of “Fold” means the magnitude of up- or downregulation for each unigene after BL exposure; “+” indicates upregulation and “-” indicates downregulation.
