# Supplementary material for: Transcriptome Sequencing and Comparative Analysis of Saccharina japonica (Laminariales, Phaeophyceae) under Blue Light Induction
Source: PLoS One. 2012 Jun 27;7(6):e39704. doi: 10.1371/journal.pone.0039704 (PMC3384632; doi:10.1371/journal.pone.0039704)
Supplement: File S12 — 2 significant differentially expressed unigenes related to blue light induced photomorphogenesis in S. japonica. (DOC) [file pone.0039704.s012.doc]

***File S12 2 significant differentially expressed unigenes related to blue light induced photomorphogenesis in S. japonica***

| **Gene ID** | **Description** | **Fold** | **P value** |
| --- | --- | --- | --- |
| Unigene1697 | COP 9 signalosome complex subunit | 1.5638 | 1.70E-100 |
| Unigene48767 | DET1 (de-etiolated 1) | 2.8415 | 9.07E-18 |

Limitations of all differentially expressed unigenes are based on P value < 0.05 and FDR ≤ 0.001which indicated the unigene was significantly altered after BL exposure. The absolute value of “Fold” means the magnitude of up- or downregulation for each unigene after BL exposure; “+” indicates upregulation and “-” indicates downregulation.
